# Making the Case for Organ‐on‐Chip Platforms in Long‐Acting Therapeutics Development

**DOI:** 10.1002/adhm.71625

**Published:** 2026-08-21

**Authors:** Charlie Gowans, Abdulwahhab Khedr, Panagiotis G. Georgiou, Nehir Arik, Ozlem Sen, Steve P. Rannard, Tom O. McDonald, Christos Tapeinos

**Affiliations:** ^1^ Division of Pharmacy and Optometry The University of Manchester Manchester UK; ^2^ Henry Royce Institute The University of Manchester Manchester UK; ^3^ Department of Materials The University of Manchester Manchester UK; ^4^ Turkish Patent and Trademark Office Ministry of Industry and Technology Ankara Türkiye; ^5^ MRC Protein Phosphorylation and Ubiquitylation Unit University of Dundee Dundee UK; ^6^ Department of Molecular and Clinical Pharmacology University of Liverpool Liverpool UK; ^7^ Center of Excellence for Long‐acting Therapeutics (CELT) University of Liverpool Liverpool UK; ^8^ Department of Chemistry The University of Manchester Manchester UK

**Keywords:** in vitro release testing models (IVRT), long‐acting therapeutics, organ‐on‐chip, translational challenges

## Abstract

Organ‐on‐chip (OoC) technologies and long‐acting therapeutics (LATs) are two rapidly advancing fields that have not yet been meaningfully integrated. This review argues that their convergence represents a significant and timely opportunity. LAT development is constrained by a preclinical toolbox that fails to capture the coupled, tissue‐dependent processes, depot formation, sustained release, interstitial transport, host response, and local clearance that govern long‐acting performance in vivo. OoC platforms can reproduce the physiological microenvironments in which these processes unfold, including barrier structure, controlled perfusion, mechanical cues, and multicellular organization, under reproducible in vitro conditions. This review first maps the scientific and translational challenges of LAT development and the mechanistic requirements any preclinical platform must address. It then shows how the core design principles of OoC technology enable tissue‐mimicking platforms that reproduce the microenvironments of the main LAT administration routes. Finally, it brings the two fields together, mapping each LAT requirement directly onto a specific OoC capability, showing that the challenges of scale and long‐term culture stability are tractable through defined mitigation strategies, and setting out how the standardization and regulatory alignment now underway can position OoC platforms as credible decision‐support tools that complement, rather than replace, existing in vitro release models.

## Graphical abstract

LATs release drug from a tissue‐resident depot over weeks to months, improving adherence, but their development is hampered by preclinical models that cannot reproduce the coupled tissue processes, sustained release, local microenvironment change, interstitial transport, immune and foreign‐body responses, and clearance that govern depot performance in the body. This review argues that OoC platforms are uniquely suited to fill this gap. By systematically mapping each long‐acting‐therapeutic requirement onto a corresponding OoC capability, it defines how purpose‐built devices can generate predictive, decision‐support data, reduce reliance on animal testing, and complement existing in vitro release tests in long‐acting drug development.

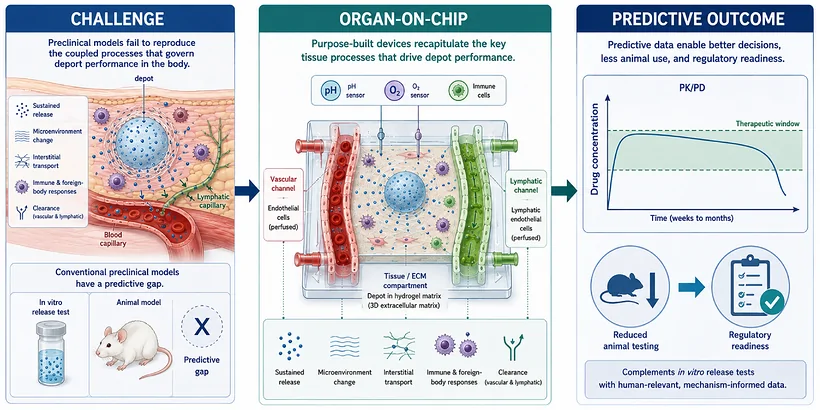

## 1 Introduction

Medication nonadherence represents one of the most persistent and costly challenges in modern healthcare. The World Health Organization (WHO) reported that 50% of patients are nonadherent to long‐term therapies [1], and a recent review found nonadherence rates in multi‐morbid patients ranging from 44.1% to 76.5% [2]. The European Commission has estimated that nonadherence costs 80–125 billion euros in potentially avoidable expenses and is associated with approximately 2 00 000 deaths annually across the European Union [3]. Conventional oral formulations contribute to this problem through frequent dosing requirements, variable bioavailability, significant first‐pass metabolism, and fluctuating plasma drug concentrations [1], all of which undermine treatment continuity. Long‐acting therapeutics (LATs) offer a fundamentally different strategy: by maintaining systemic drug exposure within therapeutic windows for days, weeks, or months from a single administration, they address the root causes of nonadherence at the formulation level [4].

Despite their clinical promise, LATs remain disproportionately difficult to develop and translate to the clinic. The core challenge is that LAT performance cannot be characterized by drug release kinetics alone; it emerges from interactions between the formulation and a dynamic, responsive tissue environment that evolves over weeks to months following administration [5]. Conventional in vitro release assays and animal models were designed primarily for short‐acting formulations, and both consistently fail to reproduce the tissue‐dependent, long‐timescale processes that govern LAT behavior in vivo [6, 7, 8]. The result is a preclinical toolbox with significant blind spots at precisely the mechanistic level where predictive data are most needed to support confident progression to clinical trials.

In parallel, organ‐on‐chip (OoC) technology has emerged as a transformative approach to recreating key features of human tissue physiology in vitro. By integrating living cells within microfluidic devices, OoC platforms can reproduce barrier structure, interstitial transport, mechanical forces, concentration gradients, and multicellular organization under controlled and reproducible conditions [9, 10]. These capabilities have enabled physiologically relevant modelling across a wide range of disease and pharmacological applications [9], and OoC platforms are increasingly recognized by regulatory agencies in the US, UK and EU as credible alternatives to animal testing in preclinical development, a shift that is now being formalized through new legislation and standardization initiatives. Despite the rapid and independent advances in both fields, however, OoC platforms have not yet been systematically applied to LAT development, a gap that constitutes a significant, largely untapped scientific opportunity.

This review makes the case for that convergence. Its contribution is not to reframe existing OoC pharmacokinetic (PK) work under a long‐acting label. Whereas most micro physiological PK / pharmacodynamic (PD) studies treat the drug as a solute entering the system from an upstream dose, a long‐acting therapeutic is itself a tissue‐resident depot whose release rate is governed by its own evolving microenvironment and the surrounding host response. This review identifies that depot‐centric problem and maps the specific mechanistic needs it creates onto the corresponding OoC capabilities. Central to this is defining what a purposefully designed OoC platform for LAT evaluation would need to achieve, not simply dynamic drug release measurement, but integrated recapitulation of depot formation, tissue transport, host response, and local clearance in a manner that generates data with genuine predictive and translational value. The review is structured in three parts. **Part A** establishes the scientific and translational context of LAT development: what LATs are, how they work, and why current preclinical models consistently fall short of capturing the mechanistic complexity required to predict their in vivo performance. **Part B** introduces OoC technology on its own terms, covering its core design principles, tissue‐mimicking capabilities, and the specific platforms most relevant to LAT administration routes. **Part C** brings these two bodies of work together, identifying where the mechanistic demands of LATs map directly onto the strengths of OoC systems, and outlining the standardization, regulatory alignment, and design priorities that would need to be addressed for OoC platforms to serve as credible decision‐support tools in LAT development.

## 2 Part A – The Scientific and Translational Challenges of LATs

### 2.1 LATs: Rationale, Formats, and Translational Complexity

To evaluate the fidelity of preclinical evaluation models, it is essential to define the mechanistic principles, physical formats, and in vivo mass‐transport barriers governing LATs. LATs are engineered delivery systems designed to continuously release active pharmaceutical ingredients (APIs) to maintain systemic or local concentrations within a defined therapeutic window above the minimum effective concentration (C_min_) and below the toxic threshold (C_max_) over extended timescales spanning weeks to months from a single dose [4]. While long‐acting oral formulations exist, this review focuses specifically on parenteral, tissue‐resident LATs, which are categorized into four dominant physical formats: pre‐formed surgical solid implants, injectable micro/nanoparticle depots, in situ‐forming implants (ISFIs), and microneedle array patches (MAPs). Within these parenteral architectures, sustained release is dictated by a complex interplay of formulation‐intrinsic mechanisms (e.g., polymer degradation, drug‐matrix affinity, burst release kinetics) and host‐tissue physiology. Once administered into the subcutaneous (SC) or intramuscular (IM) space, the local tissue microenvironment characterized by interstitial fluid flux, vascularization, lymphatic clearance, local enzymatic activity, and the dynamic development of a fibrous capsule via the foreign body reaction exerts profound control over drug dissolution and systemic absorption. Replicating this dynamic biological‐material interface outside the body represents the primary obstacle in predictive preclinical LAT development.

LATs offer tangible clinical, PK, and economic advantages over conventional daily oral or short‐acting parenteral regimens. By extending dosing intervals from days to months or even years, LATs reduce pill burden and overcome the adherence barriers inherent to chronic disease management [4]. From a PK standpoint, sustained zero‐ or pseudo‐zero‐order release blunts sharp peak‐to‐trough plasma concentration fluctuations. This smoothing effect mitigates peak‐concentration toxicities while maintaining sustained efficacy above the minimum effective concentration (Cmin) [11]. For instance, extended‐release leuprolide acetate depots (Lupron Depot; 1‐ to 6‐month in situ‐forming implants or microparticles) prevent the transient testosterone flares and adverse effects associated with frequent dosing in prostate cancer therapy [4]. Furthermore, parenteral LATs bypass hepatic first‐pass metabolism, markedly increasing systemic bioavailability for heavily metabolized drugs. In long‐acting antiretroviral therapy, the dual‐injectable combination of cabotegravir and rilpivirine (Cabenuva) demonstrates how bypassing gastrointestinal absorption and first‐pass clearance enables sustained, therapeutic plasma levels from bimonthly intramuscular injections [4].

Economically, while LAT formulations often carry higher initial pharmacy acquisition costs, they frequently reduce overall healthcare resource utilization. In schizophrenia, long‐acting injectable (LAI) antipsychotics such as paliperidone palmitate (Invega Sustenna / Trinza) significantly lower relapse rates and emergency room visits compared to daily oral equivalents, offsetting drug costs through reduced inpatient hospitalizations [4]. For high‐cost biologics and peptides, such as engineered monoclonal antibodies (ravulizumab) or long‐acting peptide depots, optimized release kinetics can minimize overall drug wastage and reduce clinic visit frequency, lowering the total cost of care [11]. Finally, in conditions where daily pill‐taking carries social stigma or serves as a constant psychological reminder of disease, such as HIV infection or psychiatric disorders, discreet long‐acting options like sub‐dermal implants (Nexplanon for contraception) or multi‐month depot injections substantially lower barriers to sustained treatment adherence [11].

Achieving these benefits, however, places considerable demands on formulation design and preclinical characterization. Unlike immediate‐release or modified‐release formulations, LATs must maintain efficacious systemic concentrations for extended periods while residing within and interacting with heterogeneous, dynamic tissues. Performance is governed by physicochemical properties of the API and the formulation, time‐dependent interplay between depot microstructure, local tissue physiology, host immune and foreign body responses, and systemic PKs [5]. These coupled processes make LAT development substantially more complex than conventional oral dosing, and, as the following section demonstrates, they expose the fundamental limitations of the preclinical tools currently used to characterize it.

### 2.2 Limitations of Current LAT Models

The complexity described above stress tests the preclinical toolbox: assays designed for short exposures are routinely repurposed for depots that persist in tissue for weeks to months, with predictable consequences for their reliability. Reviews across polymer depots, oil depots, and crystalline suspensions agree that no broadly accepted preclinical framework reliably predicts human depot performance, and robust in vitro–in vivo correlation (IVIVC) is achieved only for a narrow subset of products and conditions [6, 7, 8].

#### 2.2.1 In Vitro Release Tests Capture Chemistry, Rather Than the Depot Microenvironment

Current in vitro release testing formats prioritize analytical throughput over physiological relevance, leaving mechanistic blind spots in microenvironmental feedback loops that can govern long‐term erosion and depot reshaping. For biodegradable polymer depots, erosion is shaped by water ingress and by the acidic microclimate that develops within the matrix. These features can be muted in vitro when release media are highly buffered and receiver volumes are large. Wan et al. note that dialysis‐based methods may violate sink conditions at later time points and still yield unsatisfactory correlation when the formulation or scaling factor changes [8]. For lipid‐based long‐acting injectables (LAIs), recent reviews likewise highlight that developing predictive IVIVC remains challenging [7]. Across marketed long‐acting platforms, there are no universally accepted guidelines for selecting release conditions. Small changes in media composition, agitation, or device design can shift the apparent profile, making cross‐product comparability fragile [6, 8]. Standardization can therefore mislead when it locks in convenient conditions rather than the biophysical parameters that control depot behavior in tissue.

#### 2.2.2 Static Biological Models Compress Time and Erase Gradients

The inclusion of cells (primary or cell lines) adds biological content, but most 2D cultures and simple spheroids sit in large, well‐mixed reservoirs that flatten spatial gradients in drug, pH, and oxygen tension that normally develop around a tissue‐resident depot. Barrier assays on inserts can report permeability and junction markers, yet they still treat donor and receiver chambers as uniform pools and struggle to maintain stable phenotypes over long‐term experiments without perfusion. These limitations have driven the growing use of OoCs. Under flow, OoCs control transport and mechanical cues while preserving tissue interfaces, gradients, and multicellular organization over extended exposures [9, 10].

#### 2.2.3 Animal Models Integrate the Body, but Obscure Local Behavior

In vivo studies remain essential for whole‐body exposure and safety, but interpretation is often dominated by plasma profiles rather than the local depot niche. Industry experience with marketed LAIs highlights persistent challenges in IVIVC, local tolerability, and depot fate [6], while OoC reviews underscore the limits of animal models for predicting human drug responses [9]. Even when in vivo data are available, the local concentration gradients and tissue responses that govern depot behavior are rarely quantified at sufficient resolution.

#### 2.2.4 The Core Gap Is Mechanistic Integration, Including Lymphatic Clearance

Taking it together, current tools answer isolated questions: release tests describe drug escape under simplified conditions, static cultures report cell responses under simplified exposure, and animal studies integrate the organism but provide limited spatial resolution of the depot microenvironment. A missing piece is interstitial fluid turnover and lymphatic drainage, which shape local sink strength, immune trafficking, and the path from tissue exposure to systemic PK yet are rarely represented explicitly in conventional experimental models [12, 13].

### 2.3 Mechanistic Requirements for LAT Testing

Unlike immediate‐release (minutes to a few hours; e.g., conventional oral tablets) or modified‐release (∼8–24 h, sometimes up to ∼48‐72 h; e.g., extended‐release tablets/capsules) formulations, are designed as semi‐permanent depots in specific physiological compartments, most commonly the SC or IM space (weeks to months) [4]. Performance is governed not only by an API's properties, but by a time‐dependent interplay between formulation microstructure, local tissue physiology, host responses, and systemic PKs [14, 15, 16]. Mechanistic LAT testing must therefore go beyond “release‐versus‐time” and capture the physical, chemical, and biological processes driving API release, transport, and stability. Many conventional in vitro release tests fail because they treat release as dissolution‐controlled and neglect dynamic biological barriers and feedback mechanisms that define in vivo performance [17].

#### 2.3.1 Physicochemical Drivers of Sustained Release

**FIGURE 1:**
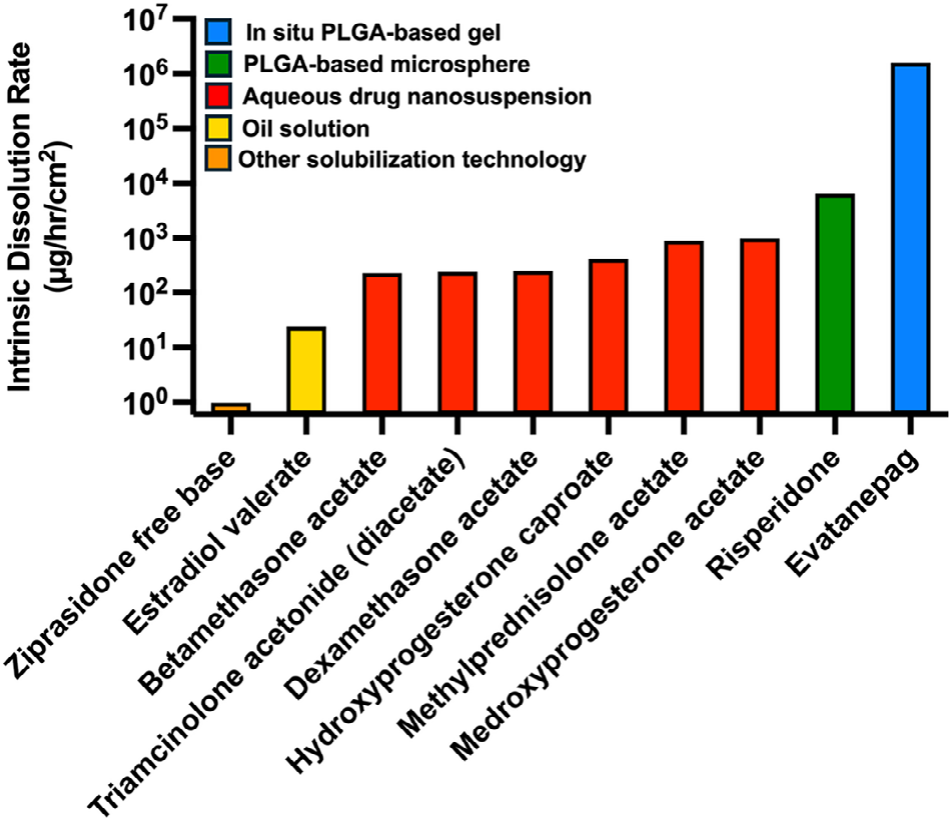
Cluster analysis correlating intrinsic dissolution rate (IDR) with long‐acting platform selection. The analysis illustrates how IDR dictates the choice of depot technology required to achieve the target in vivo PK profiles. The figure was generated using Graphpad Prism v.10 based on data reported in the literature [28].

The rate and profile of drug release from LATs are governed by formulation‐specific physicochemical mechanisms that vary across delivery platforms, including aqueous drug nanosuspensions, oil depots, biodegradable polymer matrices, and in situ forming implants [4, 18, 19, 20, 21]. Although parameters such as polymer composition [16, 22], molecular weight/dispersity [23, 24], solvent composition [25], APIs’ physicochemical properties (e.g., crystallinity, particle size, intrinsic solubility and pKa) [26], distribution, and excipient selection have been extensively reviewed [27] their behavior under physiologically relevant conditions remains less systematically addressed, particularly the influence of the host environment on release (Figure 1). Across LAT platforms, the key requirement is to achieve an absorption rate that maintains systemic exposure within the therapeutic window. Drug release and systemic absorption are distinct processes, and constant release is neither necessary nor sufficient for appropriate exposure. The PK profile instead reflects the interplay between release kinetics, local transport and clearance, drug potency, and systemic elimination. For aqueous suspensions, the intrinsic dissolution rate (IDR) is often rate‐limiting in vivo because it determines the concentration gradient that drives diffusion through the interstitium. High IDRs may require additional formulation strategies, such as microspheres or in situ gels, to prevent rapid systemic absorption [28, 29].

**FIGURE 2:**
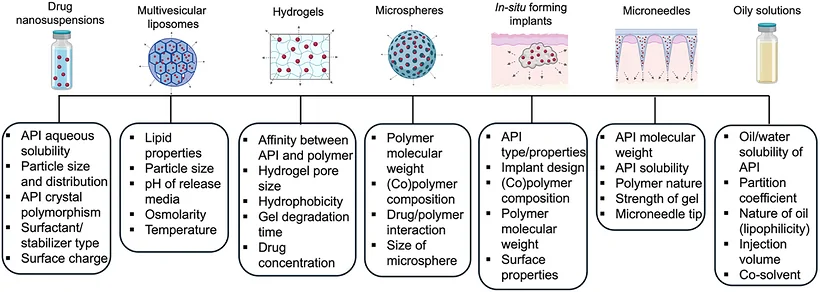
Overview of the physicochemical properties, physiological barriers, and formulation parameters that govern the release profiles of various long‐acting delivery platforms. Created with BioRender.com under agreement number EO29ZWOWXA.

In biodegradable polymer‐based systems (e.g., PLGA (poly (lactic‐co‐glycolic acid)), PLA (poly (lactic acid)), drug release is dictated by matrix mesh size, crystallinity, and water uptake, as well as time‐dependent backbone cleavage/erosion via hydrolysis or enzymatic degradation. Predictable correlation of polymer degradation and diffusional pathways is essential for moderated dissolution [16, 30]. In oil‐based depots or lipophilic solutions, release kinetics are dominated by the partition coefficient between the formulation vehicle and surrounding interstitial fluid. The interplay of API physicochemical properties, such as aqueous solubility, lipophilicity, and dose, allows interaction with the drug's terminal half‐life to dictate formulation selection. These parameters determine whether sustained systemic exposure can be achieved solely through the API's inherent properties or if dissolution‐modifying components are required to sufficiently slow the release rate. In such systems, the goal is to achieve absorption‐limited PKs, where the slow transition of the drug from the depot into the circulation becomes the primary driver of the prolonged therapeutic effect. Importantly, formulation and environmental factors that accelerate phase separation or depot solidification, such as solvent exchange kinetics in ISFIs, can substantially increase early burst release, highlighting the need for mechanistically informed testing under physiologically relevant conditions. For implantable devices, additional mechanical considerations arise [18]. Deformation, cracking, microfracture formation, or migration can create unintended interfaces for dissolution pathways. Long‐term mechanical stability testing under physiologically relevant cyclic strains, coupled with monitoring of implant‐tissue interactions, is therefore essential for accurate prediction of in vivo release behavior (Figure 2).

#### 2.3.2 API Stability and Depot Microenvironment Effects

The clinical efficacy of LATs in delivering complex biologics such as peptides, proteins, and oligonucleotides is intrinsically linked to their ability to preserve higher‐order structure and to resist aggregation, oxidation, and proteolysis. Crucially, the depot microenvironment can deviate significantly from bulk physiological conditions and evolve dynamically throughout the delivery period. Local fluctuations in pH, enzymatic activity, and osmolarity, alongside the accumulation of carrier‐derived degradation products, can compromise API stability even when cumulative mass‐release profiles appear steady. An example is observed in biodegradable PLGA/PLA‐based depots, such as those widely used for ISFIs, where hydrolytic degradation generates lactic and glycolic acid monomers. This process triggers pronounced intra‐depot acidification, which may significantly accelerate the chemical degradation of encapsulated payloads. Related stressors can occur in other depot architectures, where diffusion‐limited regions and altered water activity may promote aggregation or chemical degradation of the encapsulated payload. These challenges may be compounded in in situ forming gels, where solvent exchange and phase transition can denature sensitive biologics before sustained release begins [31].

#### 2.3.3 Biological and Physiological Requirements: Multi‐Scale Transport and Absorption

After depot release, drugs move through interstitial diffusion/convection, interact with the extracellular matrix (ECM), and enter the lymphatic or systemic circulation. Because the injection site is a responsive, heterogeneous tissue, LAT evaluation must distinguish local exposure processes from systemic disposition. A critical determinant is the depot geometry and interfacial surface area, which are shaped immediately upon administration by the interplay between formulation rheological properties and local tissue mechanics (e.g., adipose vs. intramuscular). These morphological shifts directly influence the effective release area and can create significant variability in the initial mass‐transfer rate [32]. While inter‐patient variability in gastrointestinal physiology contributes to variability in oral drug uptake, depot morphology represents an additional, formulation‐specific source of variability that arises from local tissue interactions and evolving structure at the injection site.

**FIGURE 3:**
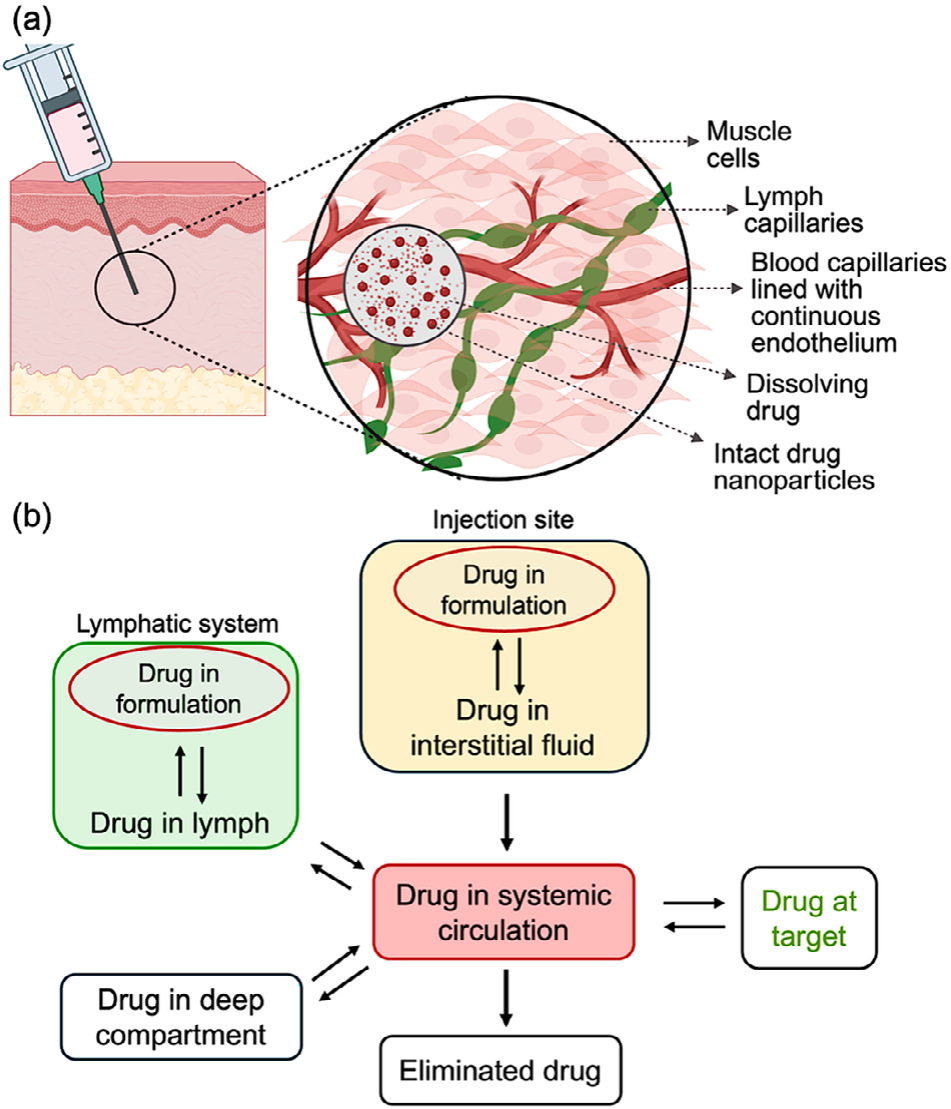
Anatomical and physiological determinants of drug disposition. (a) Schematic representation of the anatomical interface between blood and lymphatic capillaries. (b) Overview of the physiological processes governing the PK fate of lipophilic drug substances following intramuscular injection. Created with BioRender.com under agreement number FI29ZWO4KA.

Beyond initial geometry, local transport dynamics and clearance pathways dictate the long‐term PK profile. Drug movement away from the depot site typically occurs under the low‐flow, restricted‐volume conditions of the interstitium, where diffusion and slow convection dominate conditions that standard in vitro release systems rarely reproduce. The apparent release rate is further modulated by local perfusion, capillary density, and lymphatic drainage, which act as the primary filters for systemic entry. For larger biologics, lymphatic uptake adds residence time and regional enzymatic exposure [33]. Integrating these local transport measurements with physiologically based pharmacokinetic (PBPK) or compartmental models is therefore a critical mechanistic requirement; such an approach enables the translation of local concentration‐time profiles into predictable systemic exposure and clinical PD outcomes (Figure 3).

#### 2.3.4 Impact of the Host Response

Beyond passive transport, LATs elicit an active host response that dynamically reshapes release kinetics. This process follows a well‐defined cascade that shifts the rate‐limiting step from formulation‐controlled to tissue‐controlled transport. Immediately following administration, protein adsorption modifies the depot's surface chemistry and interfacial transport properties. This is followed by a cellular phase where macrophage infiltration and differentiation occur; these cells can fuse into multinucleated cells to form a granuloma around the formulation [34, 35]. These immune cells release proteases that can accelerate carrier degradation or destabilize sensitive APIs.

The final stage of this response is fibrotic encapsulation, where fibroblasts deposit a dense collagenous shell around the granuloma‐sequestered depot. This capsule creates a distinct mass‐transfer barrier, introducing additional diffusional resistance that may progressively decelerate drug release over months. Because these biological “filters” significantly alter tissue permeability and enzymatic exposure, simplified in vitro models that omit the foreign body response (FBR) frequently fail to predict in vivo performance [36, 37]. Mechanistic evaluation must therefore account for this evolving interface to ensure that therapeutic exposure is not prematurely terminated by localized degradation or sequestration. For instance, mechanistic testing strategies should incorporate immune‐competent components, inflammatory readouts, or validated surrogates where possible.

#### 2.3.5 Gaps in Current Testing Methodologies

Conventional LAT testing workflows often employ accelerated in vitro release conditions (elevated temperature or nonphysiological media) to compress multi‐month release into experimentally practical timescales. While such approaches may support batch discrimination, they are frequently not mechanistically representative of in vivo depot performance and therefore are unsuitable for establishing predictive IVIVC unless formally validated [11, 38, 39, 40]. Elevated temperatures can alter polymer mobility, phase behavior, or water uptake relative to physiological conditions, while nonphysiological media may perturb drug solubility, depot microstructure, or degradation pathways. More importantly, in vitro systems cannot reproduce tissue transport, interstitial fluid dynamics, immune responses, or local mechanical stresses that govern absorption in vivo. For LATs whose exposure profiles depend on coupled depot–tissue interactions, empirical time‐scaling without mechanistic equivalence risks generating nonpredictive data. Compounding this, no standard release testing methods are currently recommended by regulatory agencies for most LAT formats beyond LAI suspensions [41, 42].

## 3 Part B – OoC Technologies: Capabilities, Design Principles, and Emerging Platforms

### 3.1 Core Principles of OoC Technology

Conventional 2D cell cultures are cost‐effective and scalable, but they lack key features of native tissues, including multicellular organization and relevant transport gradients. On the other hand, 3D cultures better approximate tissue architecture and can improve assessment of drug penetration compared with 2D systems, but they still fall short of reproducing organ‐level function and long‐term physiological dynamics [10]. OoC systems consist of engineered or biologically derived miniature tissues cultured within microfluidic devices and could offer advantages over conventional in vitro and in vivo models for studying PKs and drug exposure of LATs [43].

Early micro‐engineered tissue systems emerged in the 1990s, including microscale constructs demonstrating that channel geometry could direct cell alignment and tissue‐like organization [44, 45]. Progress accelerated with the introduction of optically transparent elastomers such as poly (dimethyl siloxane) (PDMS), which enabled microfabrication and real‐time imaging of living cells [46]. The term “OoC” was popularized in 2010 with microfluidic models recreating tissue interfaces under flow [47] and the field has since expanded rapidly as a platform for modelling human physiology in vitro [48].

These systems recreate key physical and biological features of organs, including shear stress, concentration gradients, multicellular organization and tissue interfaces. Controlled perfusion generates physiologically relevant mechanical forces that influence cell morphology and function [49], distinguishing OoCs from static 3D organoid models. By incorporating dynamic flow, OoCs better model drug absorption, distribution, metabolism and excretion (ADME) [50]. OoC platforms can also sustain cell cultures for extended periods [51], making them potentially suitable for the long‐term monitoring of drug release required when evaluating LATs that act over several weeks to several months. Given ongoing advances in media recirculation, on‐chip biosensing and long‐term perfusion strategies, we anticipate that extending stable culture toward durations of several weeks to a few months is a realistic near‐term goal, though matching the multi‐month exposure profiles of some LATs will likely require further platform‐specific engineering and remains an important open challenge for the field [52].

### 3.2 Building LAT‐Relevant Microenvironments

Hydrogels represent a versatile platform with tunable mechanical and biochemical properties, mimicking the hydrated porous nature of the interstitial space [53]. Those developed from natural polysaccharides have been widely used as SC models for long‐term IVRT due to their long‐term stability [54]. For example, simple agarose hydrogel formats, including gel layers cast in a petri dish [55] or in a UV‐Vis quartz cuvette [56, 57] have been developed as SC IVRT models. These SC models have been used for characterizing the in vitro release of both large (e.g., insulin, from lipid implants [55] and nanosuspensions [56]), and small (e.g. naproxen nanosuspension [56] and piroxicam PLGA ISFI [57]) molecules. In several cases, these studies involved conventional subcutaneous formulations rather than true long‐acting depot systems. In the UV‐Vis cuvette format, nanosuspension formulations were mixed into agarose to form a bottom layer, followed by a layer of plain agarose and then PBS as the release medium, enabling real‐time quantification of drug release [56]. Implants were placed at the center of the gel layer to enable diffusion to the surrounding matrix. Release was monitored either by UV‐Vis imaging of the gel layer over time or by drug quantification using HPLC for gel samples taken at specified distances from the implant center. In addition, agarose thin cast gel layers have been used to form an envelope surrounding implantable PLGA/ethyl cellulose‐based formulations of different geometries (e.g., films, microspheres, and cylinders) loaded with different model drugs [58]. This was an in vitro release testing step in which agarose was used externally to physically restrict implant swelling and deformation, mimicking the tight tissue confinement observed in vivo. As a result, more sustained release from the agarose‐enveloped implants has been achieved than with the conventional release method using a dissolution medium alone [58]. Combining fluid dynamics with gel biomatrices, BioJect cells have been designed by integrating a molded agarose gel layer into a USP apparatus type IV flow‐through cell modified with an injection needle and injection hull [59]. Using modified simulated SC interstitial fluid with optimized flow rates, BioJect demonstrated improved in vitro discrimination between tested insulin formulations [59]. Although these insulin products were not long‐acting depot systems, the platform illustrates how controlled interstitial flow and tissue‐mimetic matrices can enhance mechanistic understanding of subcutaneous drug release. Incorporation of ECM‐derived components such as hyaluronic acid (HA) and collagen (COL) further increased bio‐relevance and supported improved characterization of release behavior [59].

The use of ECM‐derived materials, particularly de‐cellularized ECM (dECM) rather than inert hydrogels, for developing in vitro models can reproduce the native structure and composition of the LAT microenvironment. While these materials preserve the structural components that control drug‐tissue interactions, their wider use in such models is restricted by the complexity arising from batch‐to‐batch variability, sourcing, and handling [60]. Alternatively, soft materials based on single or mixed ECM‐derived components can yield more robust models while preserving relevant tissue characteristics. For instance, HA, a glycosaminoglycan component of the hypodermal ECM, has been used in an in vitro release model known as SCISSOR [61]. This model could enable real‐time monitoring of the physicochemical changes in test formulations and has been used to provide more precise predictions of the clinical SC fate of monoclonal antibodies. Because of HA's high aqueous solubility and its migration into the release buffer, leading to loss of its rheological properties over 72 h, it can only be used for short‐term testing of drugs with SC absorption within a few hours. Consequently, the development of more stable materials suitable for LATs testing involved the use of hybrid hydrogels based on HA and other ECM‐derived components. Cross‐linked HA has been used in SCISSOR systems with different types of COL, producing hydrogels that remained stable for ∼6 days [62]. Similarly, covalent cross‐linking of HA with COL type I resulted in a series of hybrid hydrogels with controllable viscoelastic properties, exhibiting material stiffness over the range of 1.5 to 4 kPa, satisfying the need for LATs testing [63].

Absorption pathways (i.e., vascular or lymphatic) are determined by drug‐specific physicochemical properties and anatomical differences between target tissues, and these factors have also been reflected in model design. Considering this, a biomimetic multi‐chamber microfluidic chip has been designed with an injection chamber, a 3D hydrogel‐based SC chamber with an adipocyte‐fibroblast coculture, and an uptake chamber mimicking vascular or lymphatic absorption, depending on the presence or absence of an endothelial layer, respectively [64]. Similarly, a 3D‐printed model featuring a central SC chamber flanked by vascular‐ and lymphatic‐simulating compartments, distinguished by permeable membrane surface area, pore size, and separation distance, has been designed to replicate the physiological imbalance between vascular and lymphatic uptake pathways [65].

The maintenance of long‐term stability, sterility, and physiological conditions over long‐term experiments makes method validation even more challenging. Integration of hydrogels and ECM materials with dynamic flow systems, therefore, represents the most direct route toward building the LAT‐relevant microenvironments that the convergence with OoC technology, addressed in Part C, requires.

### 3.3 Skin‐on‐Chip (SoC) for Dermal LATs

LATs delivered via dermal routes, including intradermal, transdermal and microneedle‐based systems, require careful consideration of the local skin microenvironment. The structural organization of the skin, its ECM composition, vascular and lymphatic networks, and immune cell populations can all influence drug distribution, retention and clearance following administration. Accurate modelling of these factors is therefore essential when evaluating dermally delivered LATs.

#### 3.3.1 Skin Structure (as a Barrier and Dynamic Environment)

**FIGURE 4:**
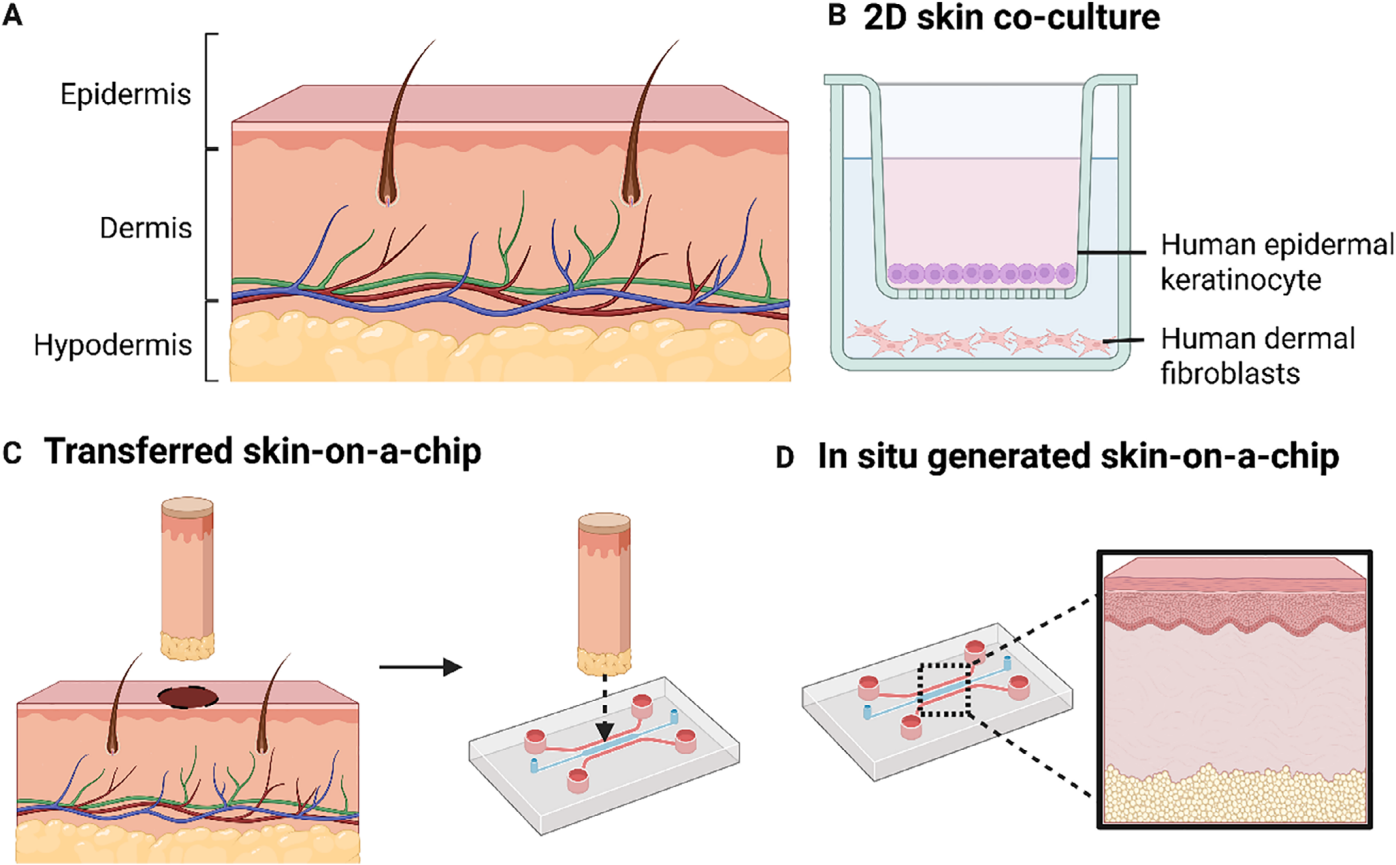
Skin structure and skin model designs: (A) A schematic diagram highlighting the three layers of the skin, the epidermis, dermis, and hypodermis. (B) A common 2D skin coculture model using keratinocytes and fibroblasts. (C, D) Schematic showing the two classifications of SoC models, transferred SoC and in situ generated SoC. Created with BioRender.com under agreement number BH2A0RTSCC.

The skin is the largest organ of the body, making up 15% – 20% of total body weight and an average external surface area of 1.8 m^2^ in adults [66]. The skin's main functions are sensory, protective, thermoregulatory, and metabolic, and it is composed of three structural layers: the epidermis, the dermis, and the hypodermis, as shown in Figure 4A. The epidermis is the outermost layer of skin and acts as an important barrier between the body and its environment, protecting from chemical, physical, and microbial damage. It also regulates the function and integrity of the underlying tissue [67]. The dermis is the middle layer of skin and, when compared to the epidermis, is relatively acellular. It is a complex system of fibrous connective tissues, composed mainly of type I and II collagens, and elastic fibers (elastin) [66], which provide the skin with tensile strength and elasticity. The dermis also hosts nerve networks, vascular networks, mast cells, macrophages, adipocytes, lymphocytes and stem cells [66]. The hypodermis is the deepest structural layer and consists of loose and well‐vascularized connective tissue that joins the skin to the surrounding organs [68]. It is mainly composed of fibroblasts, adipocytes, and macrophages [69], and contains larger nerves and blood vessels than those found in the dermal layer.

#### 3.3.2 Skin Models

The simplest approach to creating a skin model in vitro is to culture primary human keratinocytes and fibroblasts as a 2D monolayer (Figure 4B). Although these models allow for controlled investigation of cell signaling, morphology, and basic permeability, they lack the structural and functional complexity of the in vivo tissue [70]. More complex in vitro skin systems include Human skin equivalents (HSE), which are commonly produced by seeding keratinocytes onto a fibroblast‐embedded dermal scaffold and culturing initially under submerged conditions before raising to an air‐liquid interface to promote stratification [71]. Currently, there is a range of commercially available HSEs for toxicity tests, skin sensitivity tests, or drug screening, including T‐Skin (Episkin, Lyon, France), Full Thickness model (Sterlab, Saint Bernard, France), EpiDermFT (MatTek Corporation, Ashland, USA), and Phenion FT (Henkel AG & Co. KgaA, Düsseldorf, Germany) [71].

Despite their utility, both 2D and HSE exhibit key limitations. They lack a functional vascular network, limiting oxygenation, nutrient delivery, waste removal, and preventing modelling of systemic drug uptake, an essential component for LATs [72]. Static culture conditions preclude the establishment of physiological concentration gradients and do not support long‐term studies of depot degradation. Both 2D and 3D models cannot fully recapitulate injection‐site reactions, inflammation, vascular clearance or perfusion‐dependent release kinetics that occur following LAT administration. Animal models have traditionally been used to address the gaps between in vitro and in vivo, but species‐specific structural differences and the need to implement the 3Rs (Replacement, Reduction and Refinement) principles highlight the importance of more human‐relevant in vitro systems [73]. Modelling human skin in vitro*, rather than relying on animal models*, would ensure translatability of research and results and reduce animal experiments during pre‐clinical evaluations.

Skin‐on‐chip (SoC) introduces crucial features absent in conventional HSEs, including controlled perfusion, dynamic flow, oxygen and nutrient gradients, and the incorporation of endothelial, stromal and immune cells. SoC models can be roughly classified by how the skin is generated on the chip, with two approaches under development, as shown in Figure 4C,D. The first involves integrating pre‐formed tissue, either human biopsies or fully constructed HSEs. These models have been widely used in the cosmetic industry for irritation and toxicology evaluation [74]. In situ generated SoC models have been developed more recently and can be constructed with three layers, that is, epidermal, dermal, and vascular layers, separated using transparent, porous membranes, allowing interlayer communication [75].

Currently, there are no examples of SoC systems being used to investigate dermal LATs such as MAPs, although a few studies have explored SoC platforms for drug delivery more broadly Table 1. Lukacs et al. used a novel rodent transplant SoC model that utilizes continuous flow to investigate transdermal drug delivery [76]. OoC platforms have also been developed to assess microneedling‐driven skin cancer treatments, with a multi‐layer human skin/skin cancer‐on‐chip model that closely mimics in vivo skin structure [73]. If these models can be modified to investigate LATs, they could significantly enhance our understanding of the in vivo release dynamics and improve therapeutic developments.

**TABLE 1:** An overview of SoC and BBB models that can be used for modelling LAT delivery.

Model (Reference)	Species	Layers	Vascularization	LAT‐Relevant Features / Use	Ref.
3D‐printed perfused SoC	Human	Full thickness (epidermis + dermis)	Perfused microchannels (no cells)	Perfused nutrient flow through microcapillary channels; used TNF‐α and dexamethasone to test inflammatory response, showing drug absorption trends similar to human skin. Enables dynamic 3D drug screening.	[91]
Microvascularised SoC	Human	Full thickness	Microvascular network (HUVECs + pericytes)	Incorporates perfusable endothelial capillaries; vascularization enhanced epidermal stratification. Developed for systemic drug efficacy/safety testing in a human‐relevant context.	[126]
Human skin/cancer‐on‐chip	Human (incl. melanoma cells)	Full thickness (epidermis + dermis; tumor spheroid)	Vascularized (micro vessels included)	Contains differentiated epidermis, dermis and embedded melanoma. Used to deliver doxorubicin via microneedles, achieving deep penetration to tumor cells—demonstrates use of transdermal LAT (microneedle) delivery.	[73]
Ex vivo SoC (pumpless)	Human (cadaver skin)	Full thickness	No innate vessels (fluidic perfusion)	Chips a real human skin biopsy under controlled flow. Perfusion channels mimic blood supply. Enables localized depot placement and monitoring of drug diffusion through tissue—used for subcutaneous/dermal release studies.	[127]
Epidermis‐on‐chip with TEER	Human (keratinocytes)	Epidermis only (multi‐layered)	None	Microfluidic chip with stratified human epidermis and integrated TEER sensor. Used for skin irritation/toxicity testing; high barrier integrity (3 kΩ·cm^2^ TEER, >99% impermeability). Can be adapted for transdermal permeation assays.	[74]
Inflammation‐on‐chip	Human	Epidermis + fibroblast dermis + endothelial layer	Yes (HUVEC channel)	Three‐layer human skin chip (Keratinocytes, fibroblasts, endothelial cells) on porous membranes. Induces TNF‐α inflammation and tests anti‐inflammatory drug (dexamethasone) effects. Models’ injection‐site inflammation and drug responses.	[75]
Rodent ex vivo skin‐chip	Mouse/Rat	Full thickness (native skin)	None (microfluidic perfusion)	Microfluidic diffusion chamber with excised rodent skin. Provides continuous flow mimicking circulation, evaluated caffeine cream penetration versus Franz cell. Useful for preclinical LAT formulation testing and PK modelling.	[76]
Biomimetic Full‐thickness SoC	Human	Full‐thickness (fibroblast‐derived dermis + epidermis)	Perfused flow (no endothelium)	Human full‐thickness model using fibroblast‐derived ECM and inert scaffold under flow. Shows enhanced dermal protein deposition and high TEER/low permeability. Suited for long‐term release studies of dermatological drugs.	[128]
Immune‐competent epidermis‐chip	Human	Epidermis‐only keratinocytes (HaCaT + immune cells (U937)	Flow channel (no vessel)	Coculture of keratinocytes with monocyte‐derived immune cells in a perfused chip. Demonstrated improved tight junctions and sustained viability (17 days) under flow. Applicable to LATs by modelling skin sensitization/allergy and barrier impact on drug delivery.	[88]
Vascularized immune skin‐chip	Human	Epidermis + fibroblast dermis + vasculature	Yes (perfused endothelium)	Contains human keratinocytes, fibroblasts and a perfusable micro vessel. Incorporates leukocytes in perfusate to emulate inflammation. Demonstrated UV‐induced cytokine release and neutrophil migration. Can be used to assess immune responses to LATs at injection site.	[89]
Hypoxia‐enhanced BBB Chip	Human	Dual‐channel microfluidic with endothelial lumen & neural side	Perfused microchannels with fluid flow	Recapitulates tight barrier for 1 week, expresses efflux pumps and selective transcytosis of peptides/antibodies. Ideal for evaluating long‐acting biologics and BBB transport mechanisms.	[129]
Emulate CNS ‐on‐a‐Chip permeability model	Human	Two‐channel microfluidic chip	Continuous perfusion (commercial setup)	Demonstrates drug permeability trends with ten compounds correlating with in vivo/animal data. Useful for LAT compound screening and physiologically based PK modelling.	[130]
3D perfusable BBB on chip	Human	3D vascular architecture	Engineered perfusable vasculature.	Physiologically relevant 3D capillary network with perivascular cells; efflux transport measurable. Supports long‐term functional studies and drug modulation testing.	[131]
Micro engineered 3D astrocytic network BBB	Human	Microfluidic BBB with 3D astrocytic network	Flow perfusion microchannels.	Reproduces tight junctions, gene expression of transporters and receptors; allows quantification of nanoparticle/drug dynamics.	[77]
Self‐assembled stem cell BBB on chip	Human	Vascular network self‐organized in microchannels	Controlled perfusion with microfluidic pumps	Detailed protocol for creating perfusable BBB with transport measurement modalities suitable for screening of therapeutic molecules and LAT modalities in a reproducible way.	[132]

### 3.4 Barrier Chips: Blood‐Brain Barrier (BBB)

BBB OoC systems represent one of the most technically mature barrier modelling platforms in the OoC field, and their inclusion here is justified on two grounds. First, LAI antipsychotics constitute one of the most clinically established LAT classes; therefore, evaluating central nervous system (CNS) drug permeability is a necessary component of the development pipeline for this therapeutic category, even though BBB interaction is governed by intrinsic drug properties rather than by delivery format. Second, the design principles established in BBB chip development, including Transepithelial (or Transendothelial) Electrical Resistance TEER‐based monitoring of barrier integrity, controlled perfusion of multicellular interfaces, and quantitative permeability measurement, represent a methodological benchmark directly applicable to the less mature tissue‐chip platforms needed for SC and IM LAT administration sites.

#### 3.4.1 Brief BBB Structure and Function

The BBB is a semi‐permeable barrier that encompasses the microvasculature of the CNS, which controls the entrance and expulsion of molecules in the vascular compartment to the brain. The BBB impedes the entrance of most blood‐borne substances from entering the brain, and this barrier function is attributed to the unique perivascular structure, specialized by a 3D network of astrocytes communicating with endothelial cells and pericytes [77]. Kozlovskaya and Stepensky showed that typically only 0.2‐ 0.5% of injected doses pass the BBB, though this figure rises when certain nanoparticles or conjugate‐based drug delivery systems (DDSs) are used [78]. The complexity of the BBB creates a major obstacle for brain drug delivery, being the primary cause of treatment failure for brain‐bound drugs.

**FIGURE 5:**
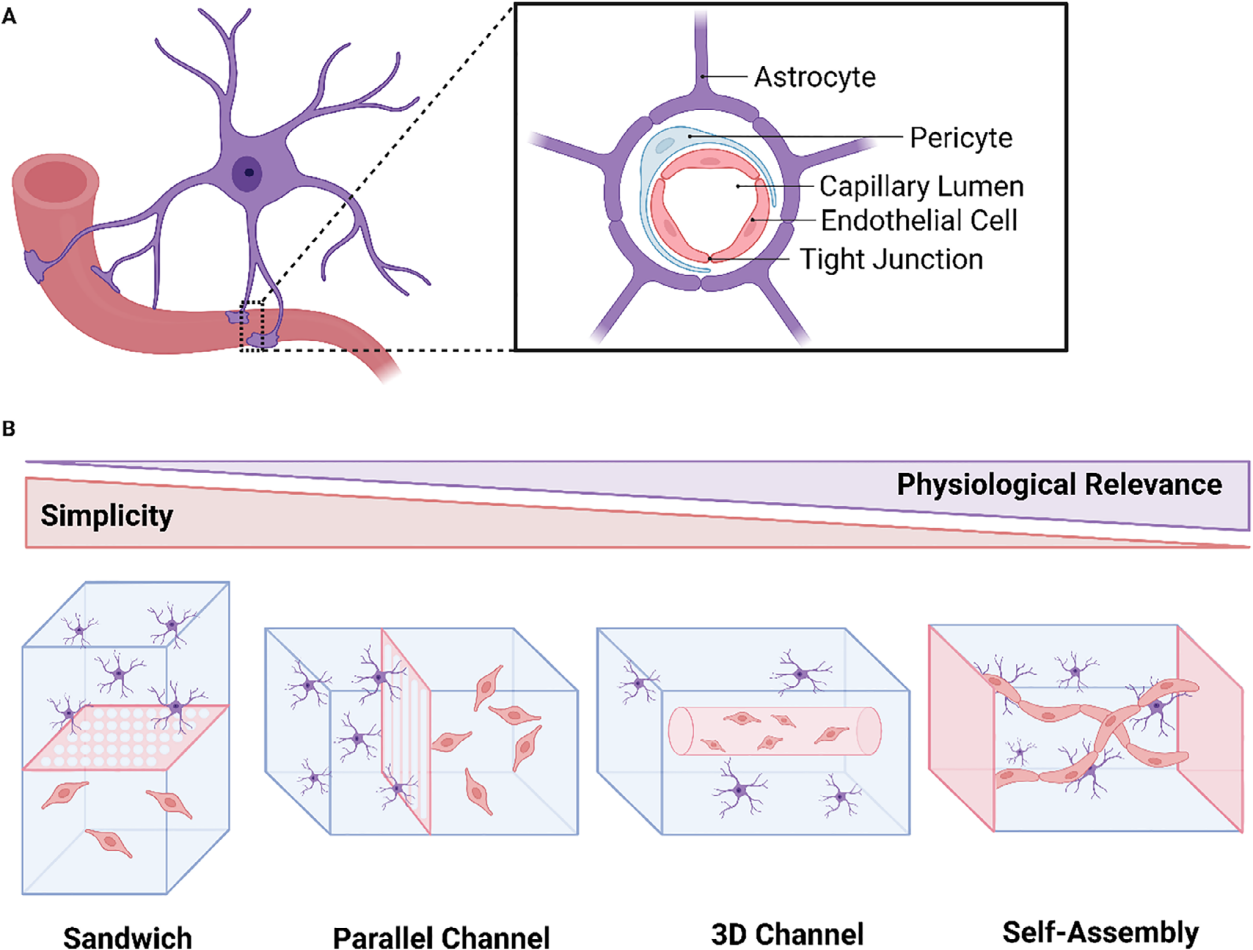
Structure of the BBB, and an overview of BBB‐on‐chip designs: (A) schematic overview of the BBB structures, the magnification window shows a cross‐section and the cellular players. (B) shows a schematic overview of BBB‐on‐chip designs, organized by their physiological accuracy and their simplicity. Created with BioRender.com under agreement number MB2A0RTKNY.

In the last few decades, multiple types of BBB models have been developed, both 2D and 3D. The earliest in vitro models were 2D cultures, easy to handle and utilized brain or brain capillary cells, they lacked many physiological components. Transwell‐based cultures allow separation of cultures by a permeable membrane, with the first reported use in the early 1980s. Although 2D approaches can be utilized for basic toxicology screening in drug discovery, they often lack the ability to predict ADME in vivo. The development of BBB‐on‐chip systems emerged in the 2000s as a powerful tool for investigating the complex biology of the BBB. There are multiple individual designs that can be categorized into four groups: sandwich design, parallel channel, 3D channels, and self‐assembly models, as shown in Figure 5. For further information, Vetter et al. (2025) provide a comprehensive review with an overview of each BBB‐on‐chip design and notable examples [79].

### 3.5 Multi‐Organ Chips for PK/PD of LATs

Interactions between organs are essential to ensure the physiological function of the human body, and although organs are physically separated in vivo, their communication via the blood and lymph circulation is vital to maintain overall viability and homeostasis. The process of ADME governs the distribution, efficacy, and potential toxicity of drug compounds once they enter systemic circulation. While long‐acting formulations are designed to control the rate of drug entry into the blood compartment, downstream organ distribution and clearance are determined primarily by intrinsic PK properties rather than delivery format. Multi‐organ models are relevant for studying systemic pharmacology downstream of LAT absorption. While the prolonged release profile is established at the depot site, the systemic consequences, including hepatic clearance, renal elimination, and target‐organ exposure, sustained over weeks to months, can only be assessed in platforms that link multiple organ compartments. In this context, multi‐OoC systems are uniquely positioned to evaluate whether the sustained plasma concentrations generated by a LAT translate into the intended PD effect at the target organ, and whether prolonged exposure carries accumulation or toxicity risks not apparent in single‐compartment models [80].

**FIGURE 6:**
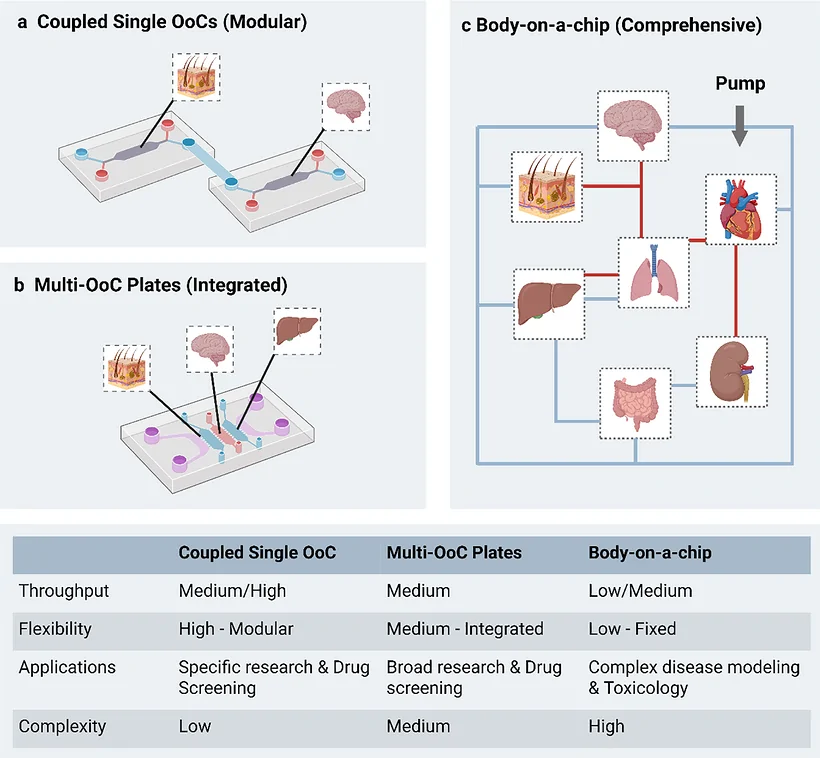
Architectures for multi–organ‐on‐chip (OoC) systems: Comparison of increasing levels of organ integration: (a) modular coupling of individual OoCs for flexible, low‐complexity studies; (b) integrated multi‐OoC plates enabling coordinated organ interactions with moderate throughput; and (c) a comprehensive body‐on‐a‐chip platform linking multiple organs via a recirculating microfluidic network for systemic disease modelling and toxicology. The table summarizes trade‐offs in throughput, flexibility, applications, and complexity. Created with BioRender.com under agreement number RG2A0RTDAP.

Multi‐organ‐on‐chip (multi‐OoC) can recreate systemic dimension and cross‐organ communication. These devices can be classified into two distinct types, coupled single OoCs or multi‐OoC plates, as shown in Figure 6. Coupled single OoCs refer to multiple chips connected via capillary tubing or a microfluidic system to reproduce systemic interactions among two or more organ models. This approach allows for multiple configurations of the whole system, as well as more individual fine‐tuning of the organ models. By contrast, multi‐OoC devices have different organ models integrated into one single plate format, with media channels acting as vascular systems to support systemic communication [80]. This approach is very similar to “human‐on‐chip” or “body‐on‐chip” systems in which almost all organs are included (Figure 6). There are advantages and disadvantages to both multi‐OoC systems, with coupled single‐OoC platforms being more useful for fundamental academic research, as they offer low‐to‐moderate throughput. By contrast, multi‐OoC plates offer higher throughput and are more suitable for preclinical, toxicity, and large drug screening. All of these systems can be optimized and integrated to test depot formation, microenvironmental differences, and transport into the blood, as shown in Table 2.

**TABLE 2:** Multi‐organ‐on‐chip devices that can be used in LAT testing and development.

Platform / Study	Organs Included	Relevance to LAT / PK Testing	Ref.
Physiome‐on‐chip	Multiple MPS (various organ models) connected as integrated system	First demonstration of interconnected multi‐organ micro physiological system designed to capture organ‐organ interactions, enabling PK/PD studies and quantitative drug fate modelling across compartments; foundational for systemic drug testing.	[133]
PEGASO multi‐OoC	Gut, immune, liver, BBB, brain compartments	Engineered a hydraulic multi‐organ platform to investigate PKs and distribution of drugs (e.g., donepezil), modelling absorption, metabolism, and diffusion across organs, directly relevant to long‐acting profile analysis.	[134]
Integrated Gut–Liver MPS	Gut + liver	Demonstrated continuous communication in multi‐organ in vitro system that simultaneously monitors intestinal permeability and hepatic metabolism, key drivers of ADME and long‐acting drug behavior in early screening.	[135]
Multi‐Organ Toxicology MPS Examples	Vascular, liver, cardiac iPSC‐derived tissues	Long‐term culture (∼28 days) with integrated biosensing enables extended observation of ADME and chronic responses, relevant to prolonged drug exposure and long‐acting formulations.	[136]
Four‐Organ Chip	Intestine, Liver, Skin, Kidney	Demonstration of long‐term (∼28 day) coculture of four human organ models with fluidic interconnection, allowing sustained ADME profiling and repeated dose systemic toxicity testing within a single platform.	[137]
Tumor + Liver Microenvironments	Tumor and liver vascular models	Series‐connected models allowed dynamic measurement of transport, accumulation and toxicity, illustrating how cross‐tissue interactions can shape drug distribution/tissue exposure profiles.	[138]
Coupled Organ Chips for PK Prediction	Gut, Liver, Kidney (and bone marrow in related work)	Fluidically linked organ chips predicted human PK parameters for orally administered nicotine and cisplatin metabolism and clearance, quantitatively matching clinical data.	[139]

### 3.6 Immune‐Competent & Stromal‐Complex Systems

LATs can sustain interaction within the skin microenvironment, which can induce an immune response in patients at the site of injection. Stromal cells in the skin are positioned in the dermal layer below the epidermis; they release growth factors, regulate inflammatory responses, and provide structural and connective support to the tissue [81]. Immune responses can lead to cellular (T cell) and humoral (B cell) reactions, including anti‐drug antibodies (ADA) [82]. The risk of ADA development can vary by patient population and therapeutic target, and the consequences can range from no effect to severe reactions impacting safety, PKs, PDs and efficacy [83]. The use of biomaterial in LAT design is the main factor affecting the severity of the immune response, so material choice to modulate the host immune response requires strategizing. This can include the selection of materials with inherent biocompatibility, loading with immunomodulatory agents, and the modification of implant interfaces with host tissue to reduce immune‐cell adhesion [84]. Alongside immune responses, it is also vital to consider local and nonspecific foreign‐body responses to LATs. Injection site reactions can include local erythema, oedema, abscess formation and acute or chronic inflammation [85]. The duration of this response can be one or two weeks and may also affect the functionality of the LATs’ PK and efficacy [86, 87]. Because of the immune response, it is critical that immune‐competent systems be included in the selection and testing of LATs.

OoC platforms can provide an opportunity to recapitulate the complex cellular crosstalk occurring at implant sites. By incorporating both stromal and immune components, OoC systems can test the release, toxicity, and immune response of LATs under conditions that closely mimic the human skin microenvironment. Recent immune‐competent OoC models have used immune cell coculture or circulating leukocytes, with Ramada and Ting coculturing immortalized keratinocytes and a human leukemic monocyte lymphoma cell line, chosen as an alternative to dendritic cells. They showed that tight junctions of the skin were improved with dynamic perfusion after 17 days of culture with online TEER measuring systems [88]. Although this immunocompetent model is considered an advanced model when compared to previous ones, a coculture of immune cells with skin cells [70] does not fully recapitulate the immune response of the skin tissue [89]. Sun et al. also reported a vascularized SoC, but using primary human neutrophils, freshly isolated from peripheral blood, and monitored the response to Herpes simplex virus (HSV) infection, as well as incorporating a fully perfusable microvascular network [90]. More recent studies have demonstrated additional advancements in SoC technologies, with a 3D perfused SoC model fabricated with projection micro‐stereolithography to create precise microcapillary‐like channels using a biocompatible resin [90]. This model was shown to exhibit drug response trends similar to human skin while showing reduced cytotoxicity over time when compared to biopsies [91]. A noncontracting vascularized full‐thickness skin equivalent model, which demonstrated relevant cytokine‐dependent immune cell recruitment and dermal response, has also been developed, providing a tool for investigating dermatological pathologies and responses [92]. The addition of immune‐competent and stromal‐complex systems can enable stronger screening of materials and design of implants, leading to reduced animal testing and improved clinical performance. Notably, immune‐competent OoC models for nondermal administration sites, including subcutaneous and intramuscular tissue relevant to injectable LAT depots, remain largely undeveloped and represent a priority gap for the field.

It is also important to note that existing immune‐competent OoC platforms largely capture the acute phase of the host response, rather than the chronic FBR that governs the long‐term performance of SC/IM depots. A dedicated FBR‐on‐a‐chip platform has been developed to model implant‐induced monocyte recruitment via cytokine gradients across an endothelial vascular‐tissue interface, and a separate hydrogel‐based 3D model has examined how substrate stiffness drives macrophage fusion into foreign body giant cells [93]. However, no published OoC system yet reproduces the full chronic FBR cascade relevant to long‐resident depots, including sustained foreign body giant cells formation, progressive collagen deposition, and fibrous capsule maturation over the weeks‐to‐month timescale typical of SC/IM implants. Until such models are established, immune‐competent OoC platforms for LAT depots should be regarded as an emerging and largely conceptual capability, rather than a validated component ready for direct integration into LAT development and screening pipelines.

### 3.7 Quantitative Readouts & Measurements

A key strategy in characterization of LATs is the ability of both in vitro release and OoC models not only to behave as qualitative tools but also to provide quantitative measurements that can detect the kinetics of depot formation/erosion and drug diffusion [41]. Robust measurement strategies could also predict the drugs’ PK/PD over long‐term studies under controlled physiological conditions [94, 95].

Measurement of drug release kinetics can be carried out simply by quantifying the drug fractions released into the release medium over time. Samples collected from the release media reservoir of the in vitro model of different methods (e.g., sample and separate, dialysis, continuous flow) [41, 96] can be analyzed for drug concentration using spectroscopic and/or chromatographic techniques (e.g., UV‐Vis, fluorescence, HPLC). Fitting drug concentration‐time curves to different mathematical kinetic models (e.g., zero‐order, first‐order, Higuchi, Peppas‐Sahlin, Korsmeyer‐Peppas, Weibull) [96, 97] can identify LAT drug‐release mechanisms by measuring specific kinetic parameters (e.g., lag time, burst fraction, diffusion and erosion rate constants) [97]. LAT‐accelerated IVRT can reduce experimental time [96]. Testing conditions such as temperature, pH, agitation rate, and surfactant level can be altered to extreme values as long as LAT stability and release mechanisms are not affected [96]. Correlation studies comparing accelerated‐to‐real‐time release data can be performed to assess the predictive power of accelerated tests.

Quantitative measurements in LAT testing can extend beyond the conventional determination of cumulative drug release to real‐time monitoring of the microstructural and morphological changes that occur during depot formation and erosion. Electron paramagnetic resonance (EPR) spectroscopy has been reported as a noninvasive method that can directly and continuously quantify the kinetics of solvent‐water exchange and polymer precipitation during ISFI formation, both in vitro and in vivo [98]. Measurement of polarity shifts using EPR is based on the presence of paramagnetic compounds that have one or more uncoupled electrons (e.g., nitroxides) as a formulation component to act as spin probes [99]. Diagnostic ultrasound is another technique that can visualize and quantify the process of implant formation, swelling, and precipitation by recording impedance changes within the implant during phase inversion [100]. In addition to the use of hydrogel matrices as release media for LAT testing, they also represent a compatible platform for real‐time imaging of depot formation and quantification of drug diffusion through the gel layer using UV‐Vis light imaging techniques [57, 101, 102]. Phase separation and polymer precipitation of PLGA ISFI in agarose gel have been recorded using a single‐wavelength visible light imaging system [101], which can be combined with ultraviolet (UV) light imaging for additional monitoring of drug release [57]. Changes in UV absorbance due to drug release and subsequent diffusion into the hydrogel interface generate “absorbance maps” that can be utilized to measure diffusion coefficients and local drug concentration gradients across the gel matrix [57, 102]. These noninvasive techniques offer spatially resolved results that can further enhance mechanistic models of in vivo depot activity.

OoC platforms allow dynamic measurements that extend beyond simple analysis of drug concentrations in the receiver compartment to capturing specific PK/PD readouts [94, 103]. Quantification of the drug in both vascular and tissue compartments of either single or multi‐organ chips can estimate the classical PK parameters (e.g., C_max_, T_max_, area under the curve (AUC), volume of distribution, clearance) through IVIVC studies [104]. Quantification of the parent drug and its metabolic compounds, as well as cellular metabolic biomarker in the culture medium as a function of time, can give insights into the rate of metabolism, as well as cytotoxicity as a PD parameter [105]. Also, TEER readouts measure barrier integrity and permeability coefficients represented by rates of solute transfer through tissue barriers [106].

With advancements in analytical techniques and microfluidic technologies, quantitative assessment of drug release, transport, metabolism, and organ functionality is now enabled by OoC systems, supporting their exploration as new approaches with the potential to reduce reliance on animal testing.

## 4 Part C – Convergence and Opportunity: Advancing LAT Development Through Mechanistically Aligned OoC Systems

### 4.1 Mapping LAT Requirements Onto OoC Capabilities: The Case for Convergence

The core argument of this review is that the mechanistic gaps identified in Part A and the platform capabilities described in Part B are complementary. Where conventional in vitro models fail LAT testing most acutely, OoC systems offer capabilities that are particularly well aligned with these gaps. The inability of current assays to reproduce localized pH shifts, water ingress, and enzymatic activity around a tissue‐resident depot corresponds directly to the capacity of hydrogel‐based and perfused OoC environments to sustain and measure these microenvironmental conditions in real time [9]. The absence of interstitial transport and lymphatic clearance from existing release models, identified in Part A as a critical mechanistic blind spot, maps onto the controlled low‐flow perfusion, ECM architecture, and compartmentalized vascular channels that OoC platforms are specifically designed to reproduce [105]. The fibrotic cascade and immune‐mediated reshaping of the depot interface, which progressively alter the rate‐limiting step of drug release over months and are entirely absent from conventional assays, can be partially recapitulated through the immune‐competent, stromal‐complex OoC configurations described in Part B [93]. The mechanical forces that reshape depot geometry at the administration site correspond to the tunable strain and compression capabilities of OoC systems that static models cannot provide.

This alignment defines both the opportunity and the design brief. An OoC platform for LAT evaluation is more than a sophisticated release vessel. It is a system engineered to reproduce the specific coupled processes that govern the LAT of interest, selected and validated against the dominant in vivo rate‐determining mechanisms rather than against convenient experimental conditions. In practice, this integration proceeds in four steps. First, the dominant rate‐determining mechanism is identified for the LAT format under study, whether intrinsic dissolution rate for aqueous suspensions, polymer erosion and intra‐depot acidification for PLGA depots, or partition and phase separation for oil depots and ISFIs. Second, the minimal set of OoC modules that reproduces that mechanism and its principal in vivo sink is selected. Third, the context‐of‐use and readouts are defined in advance, from released mass to local concentration and pH profiles. Fourth, these local measurements are coupled to a PBPK or compartmental model so that on‐chip data translate into predicted systemic exposure. Realizing this requires two enabling conditions that the remainder of Part C addresses in turn: a standardization and regulatory framework that allows data generated on these platforms to be interpreted and acted upon with confidence, and design principles that ensure OoC architecture is mechanistically aligned with the biological reality of the administration site. To make this convergence realizable, LAT‐relevant OoC systems should be defined by the in vivo process they are intended to reproduce. Table 3 maps key LAT rate‐determining processes to corresponding OoC design requirements, quantitative readouts and validation comparators. This provides a framework for judging whether a proposed OoC model is likely to add predictive value, and highlights where the field remains mature, emerging or still largely conceptual, with progress in some areas, for example local transport and tissue binding, being more developed than others.

**TABLE 3:** Mapping LAT rate‐determining processes to OoC design requirements and validation outputs.

LAT‐relevant process	Dominant in vivo consideration	OoC design requirement	Quantitative readout	Validation comparator	Current maturity
Depot formation and early burst release [62]	Solvent exchange, phase inversion, depot geometry, local confinement	Tissue‐like matrix, restricted volume, controlled fluid exchange, imaging‐compatible platform	Depot geometry, phase transition, early concentration gradients, burst fraction	IVRT, ex vivo imaging, in vivo depot morphology where available	Partially supported by hydrogel and SC models; limited LAT‐specific OoC evidence [31, 32, 53, 54, 59, 96, 97, 98, 100, 101, 102, 140].
Local transport and tissue binding	Diffusion, convection, ECM binding, restricted fluid volumes	ECM‐like hydrogel, low‐flow perfusion, tuneable matrix composition	Diffusion coefficient, tissue retention, local concentration‐time profile	In vivo absorption profiles, PBPK inputs	Emerging with commercial tools available [33, 53, 57, 59, 60, 61, 62, 63, 94, 102, 103, 140].
Lymphatic or vascular uptake	Molecular size, endothelial permeability, lymphatic drainage, capillary uptake	Vascular/lymphatic compartments with endothelial barriers	Compartmental transport rate constants, lymphatic versus vascular partitioning	Clinical bioavailability, lymphatic absorption data, PBPK/compartmental modelling	Emerging for biologics; limited for depot‐based LATs [64, 141]
Host response and foreign body reaction	Macrophage recruitment, inflammation, fibrotic encapsulation, altered permeability	Stromal and immune‐competent tissue matrix with inflammatory readouts	Cytokines, macrophage phenotype, collagen deposition, permeability change	Histology around depots/implants, local tolerability data	Largely conceptual for chronic LAT depots, wd [6, 34, 35, 36, 37, 70, 85, 86, 87, 88, 89, 92].
Sustained release and API stability within the evolving depot microenvironment	Local pH shifts and intra‐depot acidification (e.g. PLGA hydrolysis), enzymatic activity, altered water activity, evolving over the delivery period	Perfused, hydrogel‐embedded depot compartment with in‐line pH/O_2_ sensing and sampling for degradation products	Local pH and microenvironmental profile, API integrity/aggregation, carrier degradation‐product accumulation, cumulative release	IVRT with buffered versus physiologically representative media, accelerated degradation and stability assays	Sensing well established in OoC; coupling to long‐resident depot chemistry largely unexplored [16, 18, 30].
Mechanical loading at the administration site	Tissue deformation, compression and cyclic strain that reshape depot geometry, erosion and transport paths (SC vs. IM)	Tunable mechanical actuation applied to the depot–tissue interface	Depot morphology change, strain‐dependent release rate, interface integrity	In vivo depot imaging, mechanical characterization data	Mechanical actuation demonstrated in OoC; not yet applied to LAT depots [18, 32, 46].

At present, published examples in which OoC platforms have directly changed formulation or development decisions for depot‐based LATs remain scarce. However, the development and application of SCISSOR and related tissue‐mimetic in vitro platforms illustrates how more physiologically relevant subcutaneous models are beginning to support formulation screening, local transport assessment and translational prediction for injectable products [62, 107, 108]. In the future, the value of LAT‐relevant OoC platforms should be judged by whether they address a defined formulation or translational decision, as well as by whether they reproduce more biological features than conventional assays. The two principal obstacles to this integration, device‐to‐patient scaling and the gap between multi‐month delivery windows and achievable culture lifetimes, are addressed through the mitigation strategies set out in the outlook section below.

### 4.2 Standardization & Regulatory Pathways

The breadth of OoC technologies has accelerated innovation; for example, lung chips supported rapid antiviral evaluation during the COVID‐19 pandemic [109] and patient‐derived tumor microenvironment models have predicted therapeutic response ex vivo [110]. However, the diversity of OoC technologies, methods, and devices is a double‐edged sword, as it makes inter‐laboratory comparisons challenging and is a barrier to industry‐wide adoption. This challenge is compounded in biomatrix‐based systems, where variability in hydrogel composition, ECM‐derived materials, and LAT formulations further reduces inter‐laboratory comparability. Additionally, the inability to obtain large‐scale, comparable results across platforms and laboratories also limits the generation of a larger database of behaviors with defined predictive performance, which could otherwise be used to identify and determine the diagnostic specificity and sensitivity of a test [111]. These differences in testing approaches are best addressed by standardization, which is the process of developing and implementing a repeatable technical task that follows specified conditions, methods, or processes, the specifics of which were agreed upon by authority [111]. The largest authority that develops and approves standards is the International Organization for Standardization (ISO). In regulated biomedical settings, such standards underpin regulatory confidence by ensuring that data are reproducible, traceable, and comparable across laboratories and over time.

In the case of clinical trials, while specific study designs remain context‐dependent, core quality, safety, measurement, and reporting frameworks are standardized through regulation across institutions and sponsors. For example, standardized areas include manufacturing conditions (Good Manufacturing Practice (GMP)), participant safety, and data analysis and reporting requirements defined by the relevant medical regulator (e.g., the FDA in the USA or the European Medicines Agency (EMA)). Additionally, any preclinical testing used to obtain dosing and toxicity data to inform clinical trials must also follow Good Laboratory Practice (GLP). The specific standards required (often ISO standards) are guided by the specific medical regulator.

Central to these regulatory frameworks is the establishment of traceable measurement systems that ensure results can be quantitatively compared across methods, laboratories, and time. A core technical principle underpinning regulatory standardization across biomedical measurements is metrological traceability [112]. This property is defined as “*the property of the result of a measurement or the value of a standard whereby it can be related to stated references, usually national or international standards, through an unbroken chain of comparisons all having stated uncertainties*” [113]. In other words, results can be compared with an accepted reference value to quantify certainty that the result is close to the “true value” [114].

In the case of a new method, the process of establishing metrological traceability, as described by Tate and Panteghini is:

1. ″Development and characterization of suitable reference materials and their value assignment in meaningful units using reference measurement procedures,
2. Establishment of commercial routine (field) assays yielding results traceable to higher order reference materials and methods, and
3. Availability of appropriate reference intervals and decision limits″ [111].

This same reference‐based measurement logic underpins the development of biomedical standards and provides a direct conceptual framework for standardizing OoC platforms.

As established in Part B, OoCs show particular promise in preclinical testing, providing mechanistic insights into key biological processes, including disease progression and the identification of potential therapeutic targets. They also enable in vitro studies to determine efficacy and safety, including assessments of carcinogenicity, mutagenicity, and teratogenicity [115], and as such have considerable potential to reduce and replace animal testing [116]. This opportunity aligns closely with the growing regulatory demand for credible alternatives to animal models. The UK and USA have committed to phasing out animal testing as validated alternatives become established [117, 118], with the USA specifically enacting the FDA Modernization Act 2.0, which allows for alternatives to animal testing in drug and biological product applications [119]. The EU is similarly preparing a roadmap for phasing out animal tests for chemical safety assessments [120]. Collectively, these regulatory shifts increase the urgency for OoC platforms to meet formal standardization and qualification requirements if they are to serve as credible replacements for established preclinical assays.

#### 4.2.1 The Route Forward for Standardization in OoCs

Across the biomedical sector, standardization has been continually evolving from the establishment of GLP regulations in 1978 [121], to biologics and diagnostic assays [114, 122]. In these cases, the route to standardization can be generalized into the following seven stages: (1) Formally scope the standard, including definition of the intended context‐of‐use and performance requirements [111]. (2) Obtain international consensus and agreement from key stakeholders [115]. (3) Establish a directory of current reference materials and methods that have metrological traceability [116]. (4) Create a network of reference laboratories to enable interlaboratory comparison studies [117]. (5) Collaborate across the network on the development and testing of reference materials and procedures that embody the draft standard's intent, including homogeneity and stability testing [118]. (6) Iteratively draft, consult and revise candidate standards to achieve technical robustness and stakeholder consensus [119]. (7) Finalize, publish and disseminate the standard through recognized international bodies (e.g., ISO) [114, 123].

Given this established pathway, a substantial proportion of the technical standardization required for OoC systems can be built upon existing regulatory frameworks from related fields, including medical devices, in vitro diagnostics, microfluidics and cell culture. For example, there are approximately ninety established standards already mapping onto key aspects of OoC design, materials, quality control and performance assessment [116]. For OoCs, the primary barriers to qualification and regulatory uptake are the absence of harmonized terminology, device classification schemes, and context‐specific performance requirements. While many developers undertake internal technical validation of their systems, these approaches are typically ad hoc, incompletely reported, and not aligned with any agreed‐upon international qualification frameworks. This makes comparison between similar devices difficult and limits confidence in biological performance, particularly in the absence of qualification for defined contexts of use and without reference‐based measurement systems that ensure traceability across studies. It is important to note that for fast‐developing areas, it is not possible to define optimized approaches or fixed technical standards while methods continue to evolve rapidly. In this case, the introduction of minimum reporting guidelines will lead to more consistent and reproducible studies [124].

To enable standardization of OoCs, an EU focus group published a roadmap in January 2025 [23, 24]. This roadmap identified the lack of harmonized technical and performance standards as a central barrier to their widespread adoption across industry and regulation. It also emphasized the need for coordinated, internationally aligned standards covering device characterization, material biocompatibility, performance metrics and data reporting, supported through collaboration with bodies such as ISO. Importantly, the roadmap identified that standardization is a prerequisite for regulators and industry to confidently evaluate OoC systems for drug development and safety assessment. The recommendations of the roadmap translate these principles into the following technical priorities:

- Harmonized terminology, definitions and symbols should be developed across the OoC domain to ensure consistent communication and interpretation of device characteristics and performance.
- Minimum reporting requirements for cell sources, biomaterials and experimental conditions should be established, building on existing transparency initiatives to enable reproducibility and cross‐study comparability.
- Comprehensive technical standards should be created to address key aspects of OoC system design, operation and performance, including device architecture, material properties and integrated measurement approaches.
- Existing laboratory practices and regulatory frameworks should be systematically evaluated to identify where OoC‐specific standardization is required, with corresponding documentation developed to guide experimental design, data management and model qualification for defined contexts of use. This process is underpinned by standardized data structures and metadata requirements to ensure interoperability, reuse and regulatory confidence.
- Application‐specific guidance should be produced to support the deployment of OoC technologies across different domains, facilitating regulatory evaluation and promoting uptake for drug development, safety testing and personalized medicine [125].

Collectively, these developments demonstrate that the standardization of OoC platforms requires a structured translational process that mirrors established pathways across regulated biomedical measurement sciences. By establishing OoC technologies within traceable measurement frameworks, context‐specific qualification, and internationally harmonized technical standards, the field can progress from innovative research tools to regulatory‐grade platforms for preclinical decision‐making. Ultimately, effective standardization will be central to unlocking the full potential of OoCs as reliable, scalable alternatives to conventional in vitro and animal models.

## 5 Outlook and Vision

OoC technologies are now reaching a stage at which their potential value for LATs lies not in qualitative demonstration but in generating predictive, design‐relevant data that can support formulation development and translation.

Future OoC platforms for LAT evaluation should therefore treat depot formation, microenvironmental differences, transport, and clearance as an integrated system, rather than as isolated parameters. Models that decouple formulation behavior from tissue response risk producing release profiles that correlate poorly with in vivo PK/PD. In this context, OoCs should be designed to support quantitative translation to in vivo outcomes, with clear scaling assumptions and, where available, clinical PK/PD to validate predictions. This can enable early‐stage screening and de‐risking to avoid some animal studies, and later help ensure that any necessary in vivo work is reduced and more targeted.

Scale and time remain central challenges for translation. In terms of scale, long‐acting performance is highly sensitive to surface‐area‐to‐volume ratios, and a depot formed within a microfluidic device may exhibit release kinetics different from those observed in patients. OoCs intended to support LAT translation will therefore require explicit scaling assumptions, grounded in dominant in vivo mechanisms such as restricted diffusion, tissue binding, and physiological sink or nonsink conditions. Importantly, release should not be primarily driven by continuous sink conditions imposed by device design. In this regard, dialysis‐based or highly perfused systems may yield misleading results when formulation composition or scaling factors change, limiting their utility for establishing meaningful IVIVC relationships. In terms of time, many long‐acting formulations have delivery windows exceeding several months, which can be challenging for cultured cells. While OoCs are highly effective for short‐term mechanistic and exposure studies, maintaining stable, physiologically relevant cell phenotypes, device performance, and controlled low‐level drug flux over such prolonged periods remains technically demanding. Potential opportunities to work around this limitation include: 1. Using shorter‐duration OoC assays and extrapolating to longer time points using mechanistic models. 2. Developing complementary accelerated studies, where key transport or degradation processes are accelerated under controlled conditions. 3. Creating hybrid approaches that measure long‐term release profiles off‐chip and then replay these concentration–time inputs into OoCs over practical durations to decouple release from biological response. In parallel, addressing long‐term culture stability, material‐drug interactions, and chronic exposure effects will be essential for OoC systems to fully capture the pharmacology of long‐acting delivery platforms.

Another factor often missing in current OoC platforms is the role of mechanical forces at the administration site. Tissue deformation, compression, and dynamic mechanical loading can influence depot morphology, erosion, and transport pathways, particularly for injectable or implantable LAT systems. Incorporating physiologically relevant mechanical cues alongside biochemical and transport parameters will be essential for reproducing clinically relevant behavior.

Advancing the SoC and SC tissue models described in Part B toward LAT‐specific applications, including depot placement, sustained release monitoring, and immune response, remains a priority and represents the most direct route to generating physiologically relevant data for the most common LAT administration routes.

Read together, Table 3 shows that no single existing model spans more than one or two of these processes, and that OoC‐based coverage remains limited, emerging, or largely conceptual across almost every row. This is precisely the gap that a mechanistically aligned, LAT‐specific OoC platform is positioned to fill, which is what makes the convergence advocated in this review both necessary and timely.

## Author Contributions

**Charlie Gowans**, **Tom O. McDonald** and **Christos Tapeinos** contributed to the Conceptualization of this review paper. Charlie Gowans, **Abdulwahhab Khedr**, **Panagiotis G. Georgiou**, **Nehir Arik**, Tom O. McDonald and Christos Tapeinos contributed to the writing – original draft of this review paper. Charlie Gowans, Abdulwahhab Khedr, Panagiotis G. Georgiou, Nehir Arik, **Ozlem Sen**, Tom O. McDonald, **Steve P. Rannard** and Christos Tapeinos contributed to the editing of this review paper.

## Funding

This work was supported by Engineering and Physical Sciences Research Council [UKRI114] under the research and partnership hubs for health technologies.

## Conflicts of Interest

The authors declare no conflicts of interest.

## Declaration of Generative AI Use

During the preparation of this manuscript, the authors used ChatGPT (OpenAI) to assist in the conceptualization and generation of the graphical abstract. The scientific content, layout, and accuracy of the final graphical abstract were reviewed and edited by the authors, who take full responsibility for the final content. Generative AI was not used to generate, analyze, or interpret research data.

## Data Availability

Data sharing is not applicable to this article, as no new datasets were generated or analyzed.
